# Comparative Analysis of Midgut Regeneration Capacity and Resistance to Oral Infection in Three Disease-Vector Mosquitoes

**DOI:** 10.1038/s41598-019-50994-4

**Published:** 2019-10-10

**Authors:** Maria Janeh, Dani Osman, Zakaria Kambris

**Affiliations:** 1 Biology Department, Faculty of Arts and Sciences, American University of Beirut, Beirut, Lebanon; 2 Faculty of Sciences III and Azm Center for Research in Biotechnology and its Applications, LBA3B, EDST, Lebanese University, 1300 Tripoli, Lebanon

**Keywords:** Cell biology, Innate immunity

## Abstract

Mosquitoes acquire the pathogens they transmit through ingestion, and the insects’ gut constitutes the first line of defense against invading pathogens. Indeed the gut epithelium acts as a physical barrier, activates local antimicrobial peptides production and triggers the systemic immune response. Consequently, gut epithelium is constantly confronted to stress and often suffers cellular damage. We have previously shown that regenerative cells are present in the guts of adult *Aedes albopictus*, and that chemical damage or bacterial infection leads to the proliferation of these regenerative cells in the midgut. In this study, we extended the analysis of gut cells response to stress to two other important disease vector mosquitoes: *Culex pipiens* and *Anopheles gambia*e. We fed mosquitoes on sucrose solutions or on sucrose supplemented with pathogenic bacteria or with damage-inducing chemicals. We also assayed the survival of mosquitoes following the ingestion of pathogenic bacteria. We found that in adult *C. pipiens*, dividing cells exist in the digestive tract and that these cells proliferate in the midgut after bacterial or chemical damage, similarly to what we previously observed in *A. albopictus*. In sharp contrast, we did not detect any mitotic cell in the midguts of *A. gambiae* mosquitoes, neither in normal situation nor after the induction of gut damage. In agreement with this observation, *A. gambiae* mosquitoes were more sensitive to oral bacterial infections compared to *A. albopictus* and *C. pipiens*. This work provides evidence that major differences in gut physiological responses exist between different mosquitoes. The presence of regenerative cells in the mosquito guts and their ability to multiply after gut damage affect the mosquito survival to oral infections, and is also likely to affect its vectorial capacity.

## Introduction

Several mosquito species are important vectors of human and animal diseases. These include the Asian tiger mosquito *Aedes albopictus*^1^ and the urban *Culex pipiens* populations – often called the house mosquitoes. *C. pipiens* is a burden all year long and the *A. albopictus* population size has been increasing in the last decade^2^. *Anopheles gambiae* transmits malaria and is probably the most studied insect vector^3,4^. *Culex* mosquitoes are known to transmit to human several pathogens such as the yellow fever virus and filarial nematodes^5^; *A. albopictus* is a known vector for several viruses including Chikungunya, Dengue and Zika^1^. Some countries are still spared by mosquito-borne diseases, but the presence of endogenous mosquito vectors together with climatic warming and people increased mobility may dramatically change the *status quo* in the near future. Classical control strategies relying on the use of chemical insecticides often lead to the selection for resistant mosquitoes and have a negative impact on the environment^6,7^. Therefore the development of effective mosquito control methods is needed^8,9^, and understanding the insect physiology and natural defenses is a valuable element in this perspective.

Mosquitoes and other invertebrates depend on their innate immune system to fight pathogens^10,11^. Several responses have been characterized in mosquitoes including phagocytosis^12^, antimicrobial peptide production^13^, and melanization^14,15^. The mosquito gut acts as an early immune barrier: it is exposed to both symbiotic microorganisms and pathogens present in ingested food. In addition, gut cells are confronted to the immune effector molecules produced by the insect itself^16^. Consequently, the gut faces stress and possibly biological damage, which results in a massive loss of enterocytes^17^. In response, homeostatic repair pathways leading to the preservation of epithelial integrity are activated. This involves the regulation of intestinal stem cells (ISCs) that are necessary for gut regeneration. In *Drosophila melanogaster*, ISCs can proliferate quickly and massively so that enterocytes are completely regenerated in less than 60 hours in damaged midguts^17–19^.

During insects’ metamorphosis, gut larval tissue is almost entirely autolysed and replaced by adult new tissue. Some studies have focused on the guts of mosquito larvae^20^, but little information is available concerning gut regeneration in adults. Few studies reported the presence of ISCs in adult mosquitoes’ guts. The old reports were based on morphological characteristics and include a study of gut wound healing in *Aedes aegypti* and a study that revealed the presence of proliferating cells in the guts of *Culex tarsalis* after blood ingestion^21–24^. More recently, we have shown that chemical damage or bacterial infections lead to regenerative cell proliferation in the midgut of adult *A. albopictus*^25^. A similar analysis revealed the presence of adult intestinal stem cells in *A. aegypti*^26^.

In the present study, we extended the analysis of adult gut regeneration to two other important disease vector mosquitoes: *C. pipiens* and *A. gambia*e. We found that in adult *C. pipiens*, dividing cells exist in the digestive tract and that these cells proliferate in the midgut after the ingestion of pathogenic bacterial or damaging chemicals, similarly to what we previously observed in *A. albopictus*. In sharp contrast, we did not detect any mitotic cell in the midguts of *A. gambiae* mosquitoes, neither in normal situation nor after the induction of gut damage. We also show that in agreement with this observation, *A. gambiae* mosquitoes were more sensitive to oral bacterial infections compared to *A. albopictus* and *C. pipiens*.

This work provides evidence that major differences in gut physiological responses exist between different mosquito species. The presence of regenerative cells in the guts of adult mosquitoes and their ability to multiply after gut damage affect the mosquito survival to oral infections, and is also likely to affect its vectorial capacity. We expect the results of this study to have implications for vector control methods.

## Results

### General structure of the adult mosquito guts

**Figure 1:**
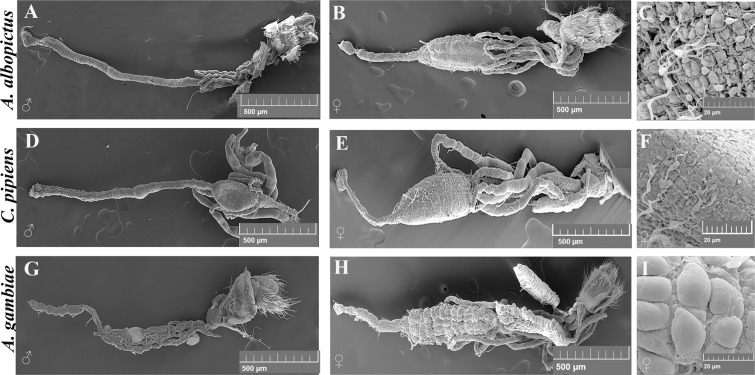
Scanning Electron micrographs (SEM) of *A. albopictus*, *C. pipiens* and *A. gambiae* mosquito guts. These photos depict the structure of female and male guts respectively as revealed by scanning electron microscopy for *A. albopictus* (**A,B**), *C. pipiens* (**D,E**) and *A. gambiae* mosquitoes (**G,H**). Two of the three gut main compartments are visible: the hindgut with the associated malpighian tubules and the midgut (arrow). Panels C, F and I are close up photos of the midgut domains.

We compared the general gut structure of *C. pipiens* and *A. gambiae* to that of *A. albopictus*. Figure 1 shows the structure of both female and male guts as revealed by scanning electron microscopy for *A. albopictus* (1A, 1B and 1C), *C. pipiens* (1D, 1E and 1F) and *A. gambiae* mosquitoes (1G, 1H and 1I). The gut epithelium is surrounded by visceral muscles and connected to tracheal branches that allow gas exchange. For the three mosquito species, only two of the three main gut compartments are visible: the hindgut with the associated Malpighian tubules and the midgut. The foregut and the crop are fragile structures that are lost during the treatment of the samples in preparation for electron microscopy. A clear difference between male and female guts is visible, male guts being overall smaller (Fig. 1A,B,D,E,G,H). This is in agreement with the fact that male mosquitoes feed on sugars only while female mosquitoes require a protein-rich blood meal to produce eggs, imposing on female guts the burden of performing more complex digestive functions. We can also note a higher degree of similarity between the guts of *C. pipiens* and *A. albopictus* females (Fig. 1B,E), while the guts of *A. gambiae* females present an anatomical structure that is more divergent (Fig. 1H).

### Proliferating cells are present in the guts of *A. albopictus* and *C. pipiens* but are not detectable in *A. gambiae* guts

**Figure 2:**
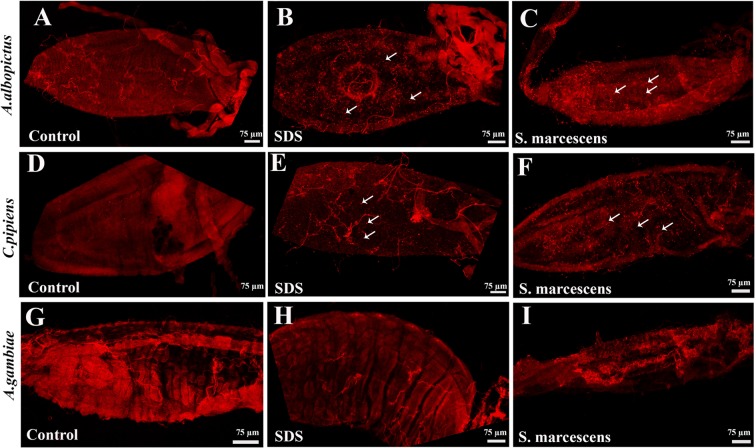
Antibodies staining for mitotic cells in the guts of *A. albopictus*, *C. pipiens* and *A. gambiae* mosquitoes. Antibodies raised against phospho-histone H3 protein (anti-PH3) show that replicative cells are present in the midguts of both *A. albopictus* (**A**) and *C. pipiens* female mosquitoes (2D). These replicative cells seem more abundant after feeding the mosquitoes on sucrose solutions supplemented with SDS (**B,D**), or with *S. marcescens* (**C**,**F**). Arrows point to representative PH3-positive cells. In contrast, no PH3-positive cells were detected in the guts of control *A. gambiae* mosquitoes (**G**), nor in the midguts of *A. gambiae* fed on sucrose solutions supplemented with SDS (**H**) or with *S. marcescens* (**I**).

Adult females of the three mosquito species were starved for two hours before being allowed to feed either on sucrose (control), sucrose supplemented with *Serratia marcescens*, or sucrose supplemented with Sodium Dodecyl Sulfate (SDS) to induce gut damage^25–28^. Mosquito guts were dissected 24 hours post-treatment and fixed. Immunohistochemistry was then performed using anti-phospho-histone H3 protein antibodies (anti-PH3), a specific marker of mitotic cells^29,30^. Similarly to what we previously reported for *A. albopictus*^25^, we observed a number of small cells with clear PH3 signal in the midguts of control *C. pipiens* midguts (Fig. 2A,D). Some PH3-positive nuclei were observed in pairs, an arrangement characteristic of two sister cells derived from the recent division of a progenitor mother cell. In contrast, the anti-PH3 antibodies were not able to detect any dividing cell in the midguts of *A. gambiae* (Fig. 2G).

### Ingestion of SDS or pathogenic bacteria increases the numbers of mitotic cells in the midguts of *A. albopictus* and *C. pipiens*

When we compared the guts of mosquitoes fed on sucrose containing either SDS or *S. marcescens* to the control guts, they appeared damaged and distorted (Fig. S3). Consistent with that, we observed an increase in the number of PH3 positive cells (Fig. 2B,C,E and F) in the damaged *A. albopictus* and *C. pipiens* guts as compared to control guts (Fig. 2A,D). These findings suggest that SDS or pathogenic bacteria feeding induces gut damage and results in the activation of local regenerative processes. In sharp contrast, we were not able to detect any mitotic cell in the midguts of *A. gambiae* mosquitoes, neither in normal situation nor after the induction of gut damage (Fig. 2H,I).

**Figure 3:**
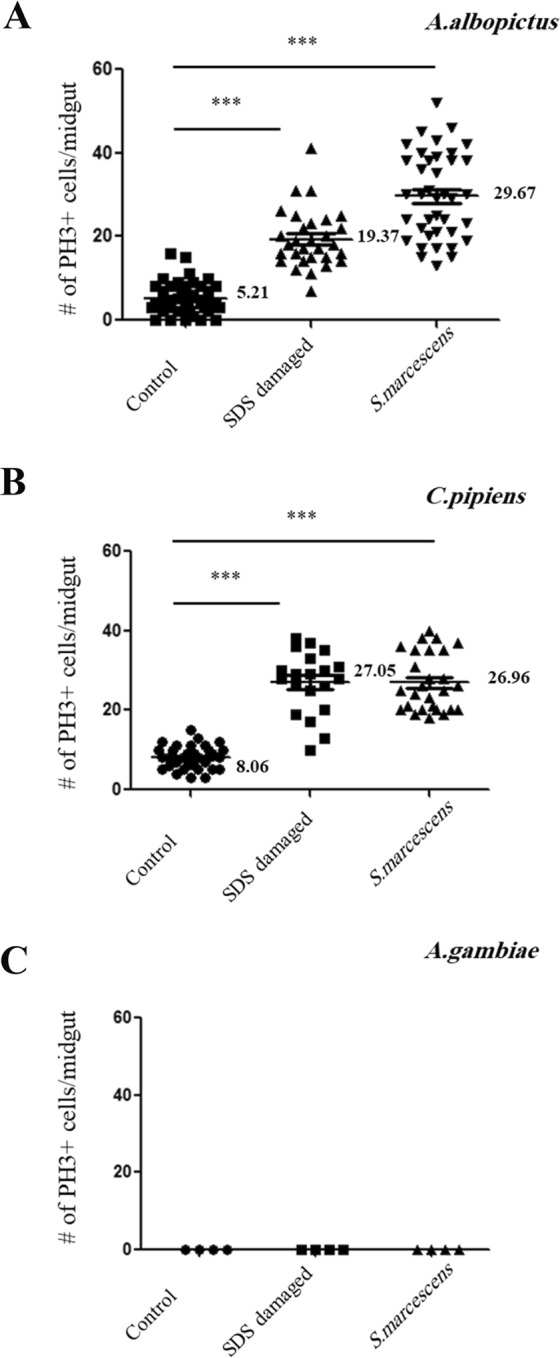
The number of mitotic cells per midgut increases significantly after damage in *A. albopictus* and *C. pipiens* mosquitoes. Feeding *A. albopictus* mosquitoes sucrose solutions supplemented with SDS induced a significant increase (p < 0.0001) in the number of replicative cells at the level of the guts (27.05 ± 1.74; n = 20) as compared to sucrose fed mosquito guts (8.06 ± 0.51; n = 33). This increase was also significant (p < 0.0001) after feeding sucrose solutions supplemented with *S. marcescens* (26.96 ± 1.39; n = 27) (**A**). *C. pipiens* mosquitoes exhibited a similar response after feeding on sucrose solutions supplemented with SDS (19.37 ± 1.26; n = 30) with a significant increase in the number of replicative cells (p < 0.0001) when compared to control sucrose fed mosquito guts (5.21 ± 0.66; n = 38). Guts of *C. pipiens* fed sucrose solutions supplemented with *S. marcescens* (29.67 ± 1.65; n = 39) also showed a significant increase in the number of mitotic cells (p < 0.0001) when compared to the control guts (**B**). This response was not observed with *A. gambiae* mosquitoes (n = 30) fed sucrose solutions supplemented with either SDS or *S. marcescens* (**C**).

We quantified the numbers of PH3-positive cells per midgut epithelium of mosquitoes fed on sucrose, SDS containing sucrose or *S marcescens* containing sucrose. For each condition, at least 20 guts were analyzed. Cell counts were plotted using the GraphPad Prism software and results are shown in Fig. 3. Feeding *A. albopictus* mosquitoes sucrose solutions supplemented with SDS induced a significant increase in the number of replicative cells at the level of the guts (27.05 ± 1.74; n = 20) as compared to sucrose fed mosquito guts (8.06 ± 0.51; n = 33). This increase was also significant after feeding mosquitoes sucrose solutions supplemented with *S. marcescens* (26.96 ± 1.39; n = 27) (Fig. 3A). Similarly, *C. pipiens* mosquitoes exhibited a significant increase in the number of replicative cells (19.37 ± 1.26; n = 30) after being fed sucrose solutions supplemented with SDS, compared to control sucrose fed mosquito guts (5.21 ± 0.66; n = 38). The guts of *C. pipiens* mosquitoes fed on sucrose solutions supplemented with *S. marcescens* also showed a significant increase in the number of replicative cells (29.67 ± 1.65; n = 39) when compared to the control guts (Fig. 3B). This response was not observed with *A. gambiae* mosquitoes (n = 30) fed sucrose solutions supplemented with either SDS or *S. marcescens* (Fig. 3C).

**Figure 4:**
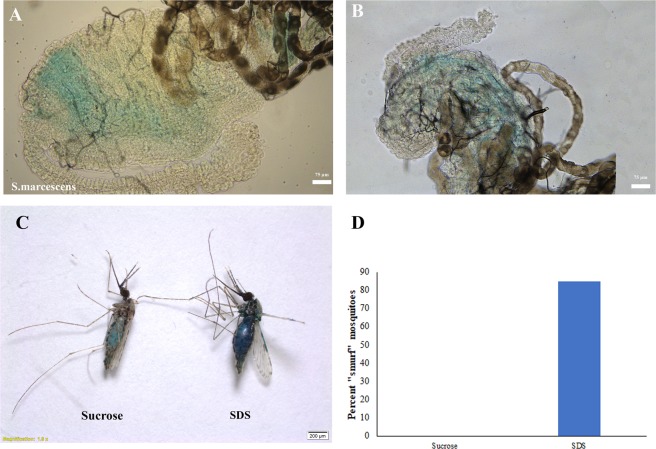
*A. gambiae* mosquitoes are ingesting the sucrose containing bacteria or SDS and are suffering gut damage. Food colorants were added to sucrose solutions supplemented with *S. marcescens* or SDS and fed to *A. gambiae* mosquitoes. 24 hours later, dissected guts revealed a blue coloration in the lumen, indicating that the mosquitoes had ingested the *S. marcescens* or SDS supplemented sucrose solutions (**A,B**). Compared to sucrose fed controls, the blue colorant diffused to different body parts including wing blades in SDS fed mosquitoes (**C**), showing a “smurf” insect phenotype as quantified in panel (**D**).

We observed similar proliferative effects when the mosquitoes were fed on sucrose supplemented with paraquat (another chemical used to induce gut stress in *D*. melanogaster)^31,32^ (Supplementary Fig. 1B,E,H) or sucrose supplemented with *Erwinia carotovora carotovora* 15 (*Ecc15*) another strain of bacteria classically used in the lab to study insect immunity (Supplementary Fig. 1C,F,I). In all cases, no mitotic cells were detected in the midguts of *A. gambiae*, despite the fact that the mosquitoes ingested the stress inducing substance (Fig. 4A,B) and that their guts were damaged (Fig. 4D and Supplementary Fig. 3). In addition, anti-PH3 antibodies successfully labeled mitotic cells in *A. gambiae* other tissues, in particular in the ovaries (Supplementary Fig. 2). We have also tried different less aggressive stress conditions (0.5% SDS, 1% SDS or 2 mM Paraquat) and examined the mosquito guts at different time points after exposure to the stressor (6, 12, 24 and 48 hours post feeding) and in all cases no dividing cells were detected in the *A. gambiae* guts (Data not shown). These results suggest that damaging the gut of mosquitoes triggers an intrinsic increase in cell proliferation in *A. albopictus* and in *C. pipiens* and that there is a major difference in the gut’s response to damage between these two mosquito species and *A. gambiae*.

### *A. gambiae* mosquitoes are ingesting the sucrose containing bacteria or SDS and are suffering gut damage

To make sure that *A. gambiae* mosquitoes fed on the damaging supplements, we added food colorant to the sucrose solution. We indeed observed blue color in their guts indicating that they did not refrain from feeding when *S. marcescens* or SDS was added to the sucrose solution (Fig. 4A,B). Compared to sucrose fed controls, the blue colorant diffused to different body parts and was clearly visible in the wing blades, in the halteres and in different lightly pigmented body parts of SDS fed mosquitoes (4 C), showing a typical “smurf” insect phenotype. This was not observed in any of the control fed mosquitoes (4D).

### Feeding on SDS leads to leaky mosquito guts and allows ingested *E. coli* to reach the hemolymph

**Figure 5:**
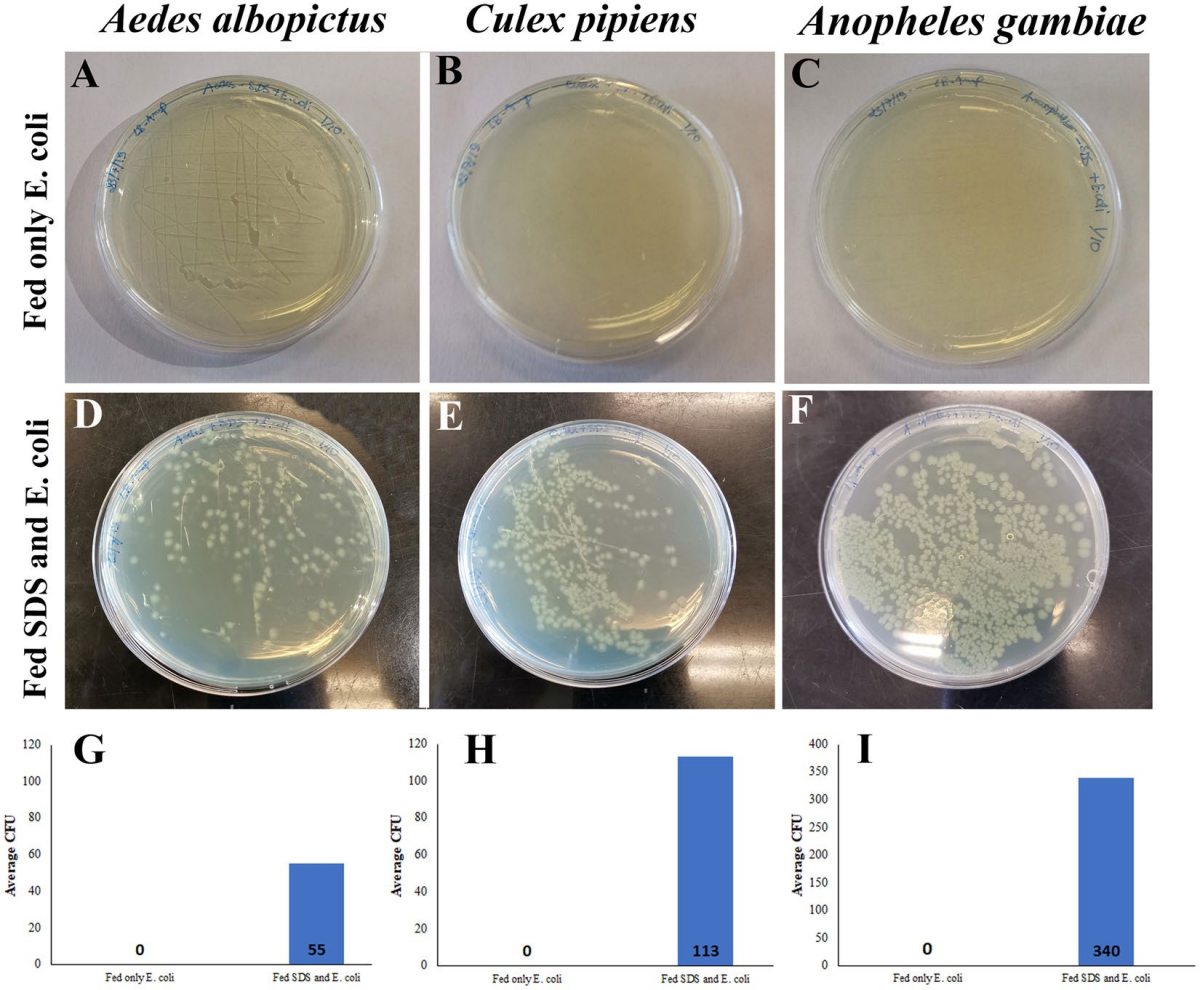
SDS treatment leads to leaky mosquito guts allowing ingested *E.coli* to reach the hemolymph. When *E. coli* was ingested by the three mosquito species, no CFUs were detected in the hemolymph of sugar fed controls (**A–C**) on the contrary to SDS fed mosquitoes (**D–F**). Panels G,H and I show the calculated average number of *E. coli* CFUs in the hemolymph of SDS treated mosquitoes.

Mosquitoes were fed on sucrose (control) or sucrose supplemented with 2% SDS for 24 hours to induce gut damage before they were offered a concentrated suspension (OD 50) of Ampicillin-resistant *E. coli* in sucrose. Hemolymph was collected from anesthetized mosquitoes and dilutions were plated on LB plates supplemented with Ampicillin. When *E. coli* was ingested by the three mosquito species, no CFUs were detected in the hemolymph of sugar fed controls (Fig. 5A–C) on the contrary to SDS fed mosquitoes (Fig. 5D–F). Panels 5G, 5H and 5I show the calculated average number of *E. coli* CFUs in the hemolymph of SDS treated mosquitoes. This experiment shows that *E. coli* is able to reach the hemolymph only after gut damage.

### Differences in mosquito survival after feeding on SDS or pathogenic bacteria

**Figure 6:**
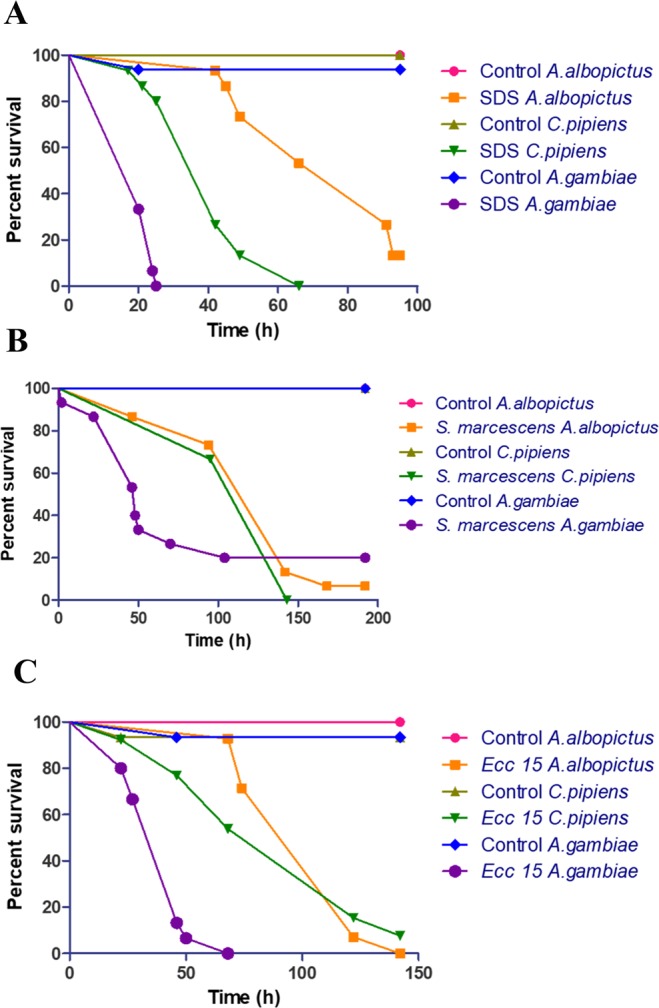
Survival of mosquitoes after feeding on sucrose solutions supplemented with SDS, *S. marcescens* or *Ecc 15*. The survival of *A. albopictus*, *C. pipiens* and *A. gambiae* mosquitoes was monitored after feeding on sucrose solutions supplemented with SDS (**A**), *S. marcescens* (**B**) or *Ecc 15* (**C**). The experiments were done in triplicates with 15 females for each mosquito species per experiment, and the rates of survivals were plotted as function of time. One representative graph is shown. *A. gambiae* mosquitoes were clearly more sensitive to the three stress-inducer supplements than *A. albopictus* and *C. pipiens*. All statistically significant differences had a p value smaller than 0.001 (p < 0.001).

The survival of *A. albopictus*, *C. pipiens* and *A. gambiae* mosquitoes was monitored after feeding sucrose solutions supplemented with SDS (Fig. 6A), *S. marcescens* (Fig. 6B) or *Ecc 15* (Fig. 6C). Feeding with SDS induced a significant decrease in the survival of mosquitoes when compared to control sucrose fed mosquitoes in all three species (Fig. 6A). When we compared survival rate amongst the three mosquito species, *A. gambiae* mosquitoes showed the most compromised survival after SDS challenge, and this increased susceptibility was statistically significant when compared to both *A. albopictus* or *C. pipiens*. The difference in survival between *A. albopictus* or *C. pipiens* after SDS challenge was also significant, with *A. albopictus* mosquitoes surviving better. Similarly, *S. marcescens* feeding induced a significant decrease in the survival of mosquitoes when compared to control sucrose fed mosquitoes for all three species (Fig. 6B). *A. gambiae* showed the highest mortality when compared to *A. albopictus* or *C. pipiens*, whereas the difference in survival between *A. albopictus* and *C. pipiens* was not significant (p = 0.1739). Similar results were observed after *Ecc15* feeding when we compared survivals of the three mosquitoes fed with the bacteria to their control sucrose fed mosquitoes: *A. gambiae* mosquitoes showed strongly compromised survival when compared to the two others, while the difference in survival between *A. albopictus* and *C. pipiens* was not significant (p = 0.6544). Altogether, the survival assays indicated that, among the three mosquito species studied, *A. gambiae* were the most fragile.

## Discussion

Due to their ability to transmit various diseases to their vertebrate hosts, mosquitoes represent a serious health threat for humankind. Indeed, it is estimated that approximately one fifth of all people dying of an infectious disease, are dying due to a vector transmitted one^33^. Mosquito-transmitted pathogens and parasites complete part of their life cycle in the insect midgut^34,35^. The midgut is not a passive structure that allows the easy development or passage of pathogens. It constitutes a very hostile environment to the invaders where they are confronted to mosquito-encoded barriers and effectors molecules that can restrict their development^36,37^. For example, *Plasmodium* suffers from the *A. gambiae* response and the number of ookinetes is severely reduced at the level of the mosquito midgut, which creates a bottleneck effect at this stage of the parasite^38^. The understanding of the cellular and molecular mechanisms that are activated in order to maintain mosquitoes gut homeostasis can be useful for the elaboration of novel control strategies of diseases vectors. It is clear however that our current data on mosquito gut regeneration will not lead to direct vector control methods, but could help on the long term to elaborate strategies such as replacing a mosquito population with another (having different gut regeneration capacity) that is refractory to certain pathogen.

The abundant and powerful genetics tools available in *Drosophila* are lacking in mosquitoes. Often scientists rely on findings and extrapolate results in the analysis of other dipteran: for instance *D. melanogaster* was a landmark model for insect immunity. The mechanisms controlling gut homeostasis have been an active topic of research in the last few years^39^ and this can certainly contribute to the understanding of how gut integrity is maintained in mosquitoes. This approach however has its limitation, and it is useful to study certain processes directly in organisms of interest, especially that the feeding habits of *Drosophila* are different from those of hematophagous insects such as mosquitoes. Previous studies have provided morphological description of the gut of some mosquito species^40–43^. We have recently investigated the regeneration of the adult *A. albopictus* guts in response to chemical or bacterial damages. We demonstrated the existence of small mitotic cells in the midgut of that have the characteristics of intestinal stem cells^25^. These results were also observed in another closely related mosquito *A. aegypti*^26^.

In the present study, we extended the analysis of gut cells response to stress to two other important disease vector mosquitoes. For the three mosquito species, we observed a difference in gut size and proportions between males and females, but this difference is expected due to the different diets of males and females. We found an important dissimilarity between the different mosquitoes’ response to gut damage: we did not detect any mitotic cell in the midguts of *A. gambiae* mosquitoes, neither in normal situation nor after the induction of gut damage, while in *C. pipiens*, dividing cells exist in the digestive tract and proliferate in the midgut after bacterial or chemical damage, similarly to what was previously observed in *A. albopictus*^25^ and *A. aegypti*^26^.

Different mosquitoes could possess different number of ISCs cells in their reservoir. An aggressive treatment (ingestion of SDS or high number of pathogenic bacteria) may lead to massive intestinal cell death and the depletion of progenitor cells in species/strains with a low original number of progenitor cells, which could make the response to the stress undetectable. It is true that based on our methods (PH3 labeling) we cannot exclude that quiescent ISCs are present in *A. gambiae* guts. However, we tried different less aggressive stresses (0.5% SDS, 1% SDS, 2 mM Paraquat) and we tried different time points (6, 12, 24 and 48 hours after feeding the stressor) and in all cases no dividing cells were detected in the *A. gambiae* guts (Supplementary Material and data not shown). Also, in contrast with what we observed in *C. pipiens* and *A. albopictus* and with what has been observed in *A. aegypti*, even in absence of damage dividing cells were not detected *A. gambiae*. Unlike *Drosophila*, in mosquitoes cell markers and transgenic lines are not available which does not help the analysis of ISCs death. In addition, even in *Drosophila* midgut apoptosis assays reveal the presence of dying cells without the ability to distinguish between different cell types (progenitors versus differentiated cells).

We showed that *A. gambiae* mosquitoes are more sensitive to oral bacterial infections when compared to *A. albopictus* and *C. pipiens*. We propose that the high sensitivity of *A. gambiae* mosquitoes to oral infections is probably due – at least partly- to the incapacity of *A. gambiae* to activate cell division to repair gut damage. In agreement with this idea, the non-pathogenic *E. coli* or food colorant was able to reach the hemolymph in the three species of SDS fed mosquitoes. Taracena *et al*. suggested that fast midgut regeneration is a contributing factor to the refractoriness of certain strains to arboviruses infection, while permissive strains lack the capacity to quickly activate the program of gut cell division^26^. It will be interesting to examine this by comparing gut regeneration of different *A. gambiae* strains, especially that the G3 strain we studied is known to be permissive to *Plasmodium*^44,45^. A previous study by Gupta *et al*. comparing the response of gut epithelium of *A. aegypti* and *A. stephensi* to *Plasmodium* infection showed different responses in these two disease vectors. This work is in agreement with our findings and corroborates the hypothesis that different species respond to gut damage differently^46^.

Similarly to our previous results in *A. albopictus*, we found that Keren (EGFR pathway) and SOC36E (Jak/Stat pathway) are induced in the guts of *A. gambiae* post SDS treatment. The induction of components of EGFR signaling pathway after *Anopheles* gut damage is in agreement with previous reports^47^. However it is known that EGFR can play a role in the morphogenesis and sloughing of enterocytes in *Drosophila*^48^ and the Jak/Stat pathway could play a role in enterocytes to modulate gut immunity (by controlling the expression of the Anti Microbial Peptide Dro3) in addition to its role in epithelium renewal^49^. Therefore, at this level, we cannot confirm which response the induction serves (homeostasis/immunity/enterocyte morphogenesis).

## Conclusion

Our study shows that major differences in gut physiological responses exist among different mosquito species. The presence of regenerative cells in the mosquito guts and their ability to multiply after gut damage probably affect the mosquito survival to oral infections and could also affect its vectorial capacity. These results, with a more in depth characterization of mosquito’s immune responses, and with an analysis of the genetic pathways that control the differences between several mosquito species should contribute to the development of alternative control strategies of theses disease vectors. These findings provide information that could be valuable for the utilization of insects as model organisms for human gut diseases^50,51^.

## Material and Methods

### Mosquito strains and rearing conditions

All animal procedures were carried according to protocols approved by the Institutional Animal Care and Use Committee (IACUC) at the American University of Beirut, and all methods were carried out in accordance with relevant IACUC guidelines and regulations.

All mosquito strains were reared in the insectary at 28 °C and 70% humidity using a 12:12 light:dark photocycle. Adults were continuously supplied with cotton pads soaked in a 10% sucrose solution and had access to water cups containing clean tap water. Larvae were fed on yeast for the first 24 hours then on fish pellet food till pupation. Pupae were collected with a plastic pipette and placed in water cups inside plastic cages.

A local strain of *A. albopictus* mosquitoes^2^ (originally captured from Sarba in the suburbs of Beirut, Lebanon) was used in this study. This strain has been kept in the insectary for more than 5 years. Feeding was allowed on anesthetized mice and eggs were collected on filter paper four days after the blood meal. Eggs were dried for two weeks before hatching was attempted by immersion in aged tap water.

A local strain of *C. pipiens* mosquitoes (Makhoul strain^52^ captured from the AUB neighborhood, Beirut, Lebanon) was used in this study. This strain has been kept in the insectary since 2014. Egg rafts were collected once every generation and allowed to hatch in tap water.

The G3 strain of *A. gambiae*, a lab colony established initially from mosquitoes collected in Gambia (Vectorbase), was used in this study. After blood feeding eggs were collected and allowed to hatch directly in the collection cups.

### Bacterial strains

The bacterial strains used in this study were *Serratia marcescens* pGEN222, *Erwinia carotovora carotovora 15* (*Ecc15*) and *Escherichia coli* DH5 alpha Ampicillin-resistant (*E. coli*).

### Scanning electron microscopy

Midguts were dissected and incubated for two hours at room temperature using a PBS fixative solution containing 25% glutaraldehyde and 4% parafolmadehyde. After three 5 minute washes in 1X PBS, the guts were dehydrated using increasing concentrations of ethanol in the following steps: 2 hours in 30% ethanol, overnight in 50% ethanol, 6 hours in 70% ethanol and finally overnight in 100% ethanol. The guts were then dried in a critical point dryer (EMS Quorum 850), coated in gold and observed under the MIRA3 LM TESCAN scanning electron microscope (SEM High Voltage: 15 kV, Detector Oxford Instruments X-Max: SE).

### Chemical and bacterial treatments

For this and all other experiments mosquitoes were between 5 and 7 days old. Mosquitoes were starved for 2 hours before their cups were supplemented with cotton pads soaked in 10% sucrose (for controls), 2% SDS in 10% sucrose, or 4 mM paraquat (Sigma-Aldrich, USA) in 10% sucrose, or a bacterial suspension OD = 50 (corresponding approximately to 4 × 10^10^ cells/ml) in 10% sucrose (for infection experiments). Less aggressive stresses were also attempted (0.5% SDS, 1% SDS or 2 mM Paraquat) and mosquito guts were examined at different time points after exposure to the stressor (6, 12, 24 and 48 hours after feeding). The mosquitoes were allowed to feed continuously until the guts were dissected for immunohistochemistry 24 hours after the beginning of the treatment.

### Isolation of mosquito midguts

Mosquitoes were cold anesthetized by placing the cups on ice, and transferred one at a time onto a glass slide in a drop of 1X PBS. Isolation of midguts was performed under a light stereomicroscope. Using fine forceps, the animal head was cut and the mosquito abdomen was pulled from the posterior end until the midgut detaches. The isolated midguts were then placed in 1.5 ml eppendorf tube containing 1X PBS and kept on ice.

### Fixation and staining

Guts were dissected and fixed for 30 minutes using a 4% Parafolmadehyde (VWR, USA) solution in 1X PBS. Then, three 15-minute washes in PBS-Triton 0.1% were performed to permeabilize the guts. After this, blocking was done for 30 minutes by adding a solution of 1X PBS -Triton 0.1%-BSA 1%, and the primary rabbit α-PH3 antibodies (ABCAM, UK) were added (1:800 in 1X PBS-Triton 0.1%-BSA 1%) overnight at 4 °C. Following three 15 minute washes in PBS-Triton 0.1%, the samples were exposed to secondary antibodies Alexa Fluor® 555 (ABCAM, UK) (1:1000 in PBS-Triton 0.1%-BSA 1%) for three hours at room temperature. Phalloidin coupled to Alexa Fluor® 647 (ABCAM, UK), was added for one hour at room temperature (1:500 in PBS-Triton 0.1%-BSA 1%). Finally, three final washes in PBS-Triton 0.1% were performed and the guts were mounted on microscope slides in anti-fade medium (Immu-Mount, Thermo Scientific).

### Fluorescent microscopy, cell counting and statistical analysis

The slides prepared were observed under an inverted fluorescence microscope (Zeiss Axiovert 200, Source: AttoArc2 HBO 100 W) for the counting of proliferating cells and an upright fluorescence microscope (Leica DM6 B) for image acquisition using the image stitching option. Cell counts were analyzed using the Graphpad Prism software and an unpaired t test was performed.

### CFU assays

Mosquitoes were fed on sucrose (control) or sucrose supplemented with 2% SDS for 24 hours before being starved for 2 hours and placed on a suspension of Ampicillin-resistant DH5 alpha *E. coli* (OD 50) in sucrose for another 2 hours. The bacterial suspension was then replaced by sucrose and 6 hours later hemolymph was collected from anesthetized mosquitoes (after clipping their proboscis) into 1xPBS containing protease inhibitor cocktail (Roche). Dilutions in sterile LB of approximately 5 µl of hemolymph were plated on LB plates supplemented with Ampicillin (100 µg/mL). The colonies were counted to estimate the approximate CFUs per mosquito.

### Survival assays

Female mosquitoes were starved for 2 hours before their cups were supplemented with cotton pads soaked in 10% sucrose for controls or in a bacterial suspension (OD = 50) in 10% sucrose. Dead insects were counted at different time intervals. Each infection was done in triplicates with 15 females for each mosquito species per experiment, and the rates of survivals were plotted as function of time. For statistical analysis of the survival data, Gehan-Breslow-Wilcoxon test was performed.

## Supplementary information

Supplementary Info
