# Supplementary material for: Comparative Analysis of Midgut Regeneration Capacity and Resistance to Oral Infection in Three Disease-Vector Mosquitoes
Source: Sci Rep. 2019 Oct 10;9:14556. doi: 10.1038/s41598-019-50994-4 (PMC6787257; doi:10.1038/s41598-019-50994-4)
Supplement: Supplementary file 1 — Supplementary Info [file 41598_2019_50994_MOESM1_ESM.pdf]

Comparative Analysis of Midgut Regeneration Capacity and Resistance to Oral Infection in Three Disease-Vector Mosquitoes

Maria Janeh[1] (msj08@mail.aub.edu), Dani Osman[2] (dani.osman@ul.edu.lb) and Zakaria Kambris[1 *] (zakaria.kambris@aub.edu.lb)

[1] Biology Department, Faculty of Arts and Sciences, American University of Beirut, Beirut, Lebanon

[2] Faculty of Sciences III and Azm Center for Research in Biotechnology and its Applications, LBA3B, EDST, Lebanese University, 1300, Tripoli, Lebanon

* for correspondence: zakaria.kambris@aub.edu.lb

Supplementary Material

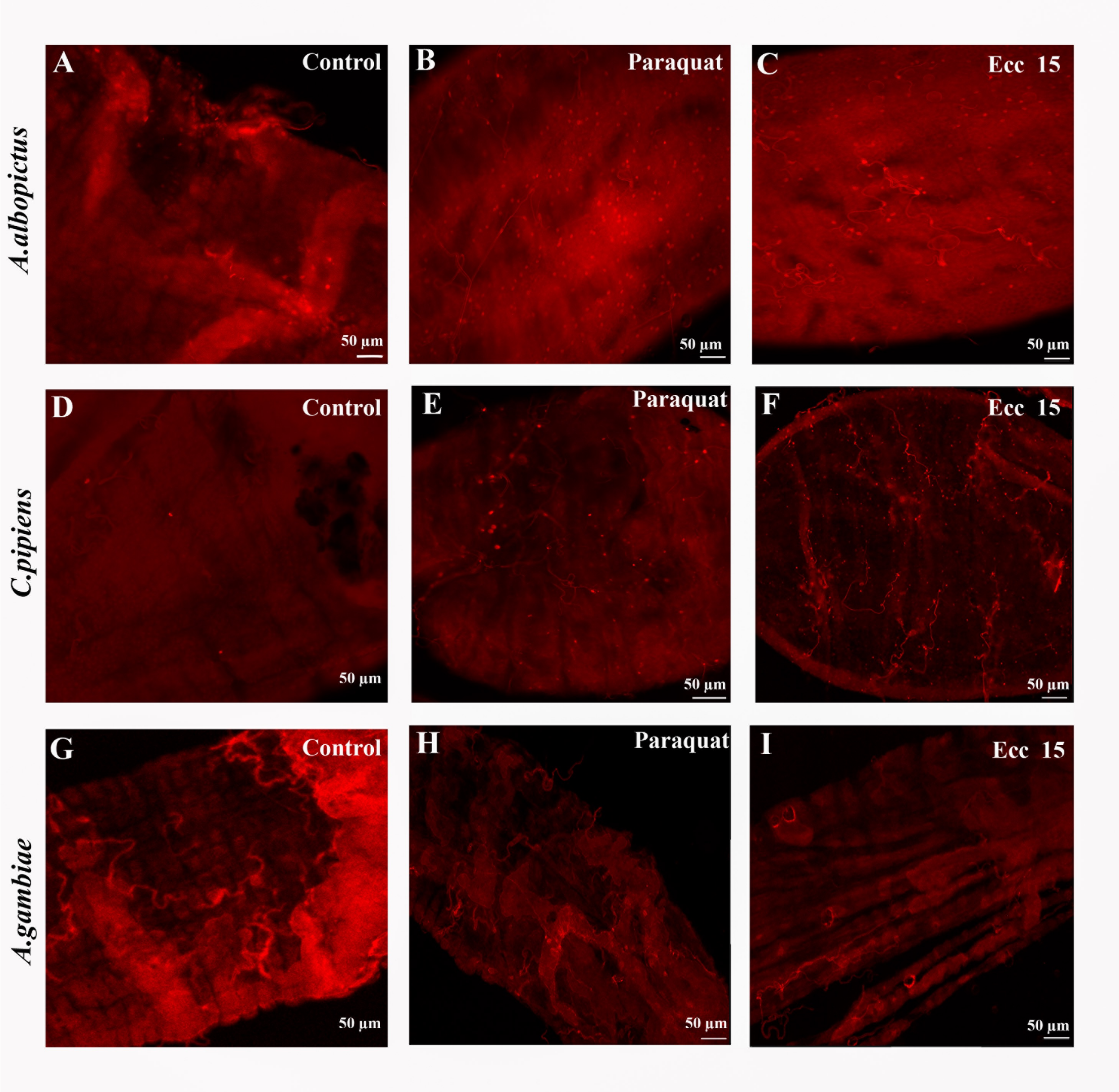

Figure S1

Figure S1: Immunohistochemistry staining reveals the presence of regenerative cells at the level of *A. albopictus* and *C. pipiens* mosquito guts

Feeding *A. albopictus*, *C. pipiens* and *A. gambiae* mosquitoes sucrose solutions supplemented with paraquat or *Ecc 15* confirm the results obtained with SDS and *S. marcescens* feeding. Immunohistochemistry staining using anti-PH3 antibodies reveals the presence of regenerative cells at the level of *A. albopictus* mosquito guts fed sucrose solutions supplemented with paraquat (S1B) or with *Ecc 15* (S1C) as compared to control sucrose fed mosquito guts (S1A).. The same results are observed with *C. pipiens* mosquito guts fed sucrose solutions supplemented with paraquat (S1E) and *Ecc 15* (S1F) when compared to the control sucrose fed mosquito guts (S1D). Staining using the anti-PH3 antibody does not reveal the presence of regenerative cells at the level of control *A. gambiae* guts (S1G), paraquat fed mosquito guts (S1H), or *Ecc 15* fed mosquito guts (S1I).

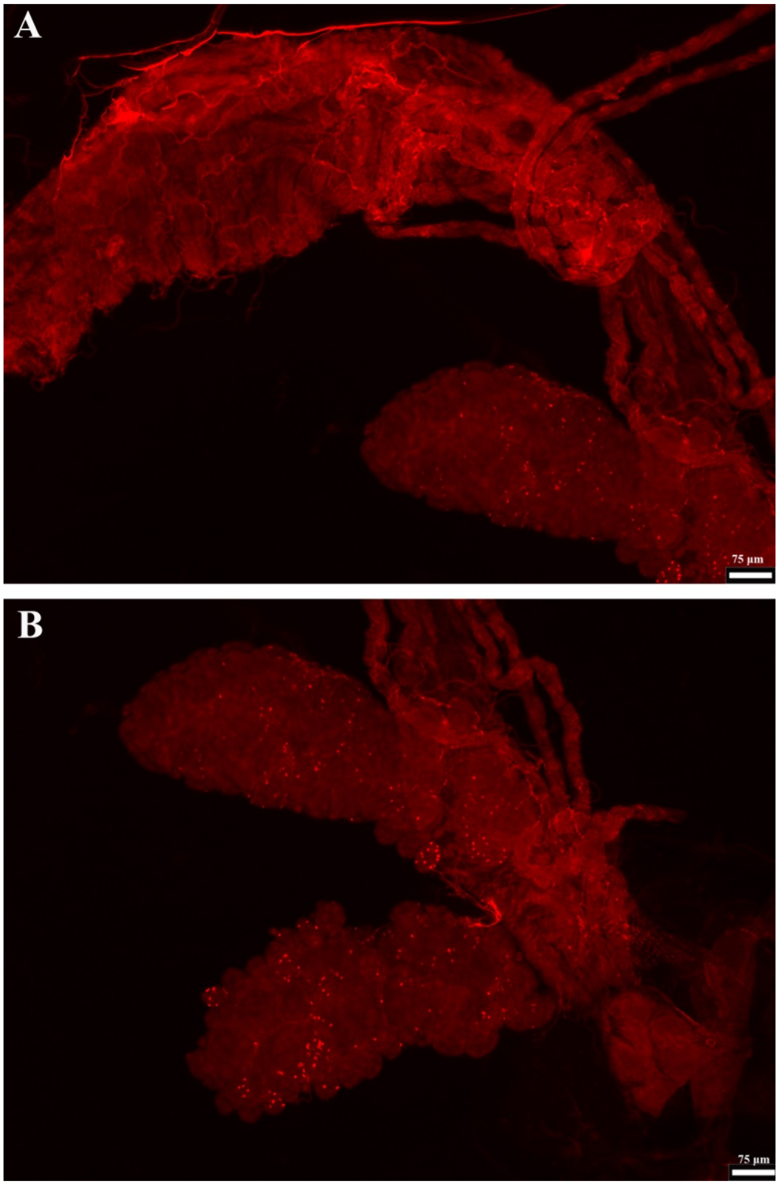

Figure S2

Figure S2: Detection of mitotic cells at the level of *A. gambiae* ovaries using anti-PH3 antibodies

Immunohistostaining of *A. gambiae* guts and ovaries using anti-PH3 (S2A) reveals the presence of regenerative cells at the level of ovaries confirming that these antibodies are functional in this species but not detecting any cells at the level of the guts. Figure S2B shows a magnification of a pair of *A. gambiae* ovaries stained with the anti-PH3 antibodies.

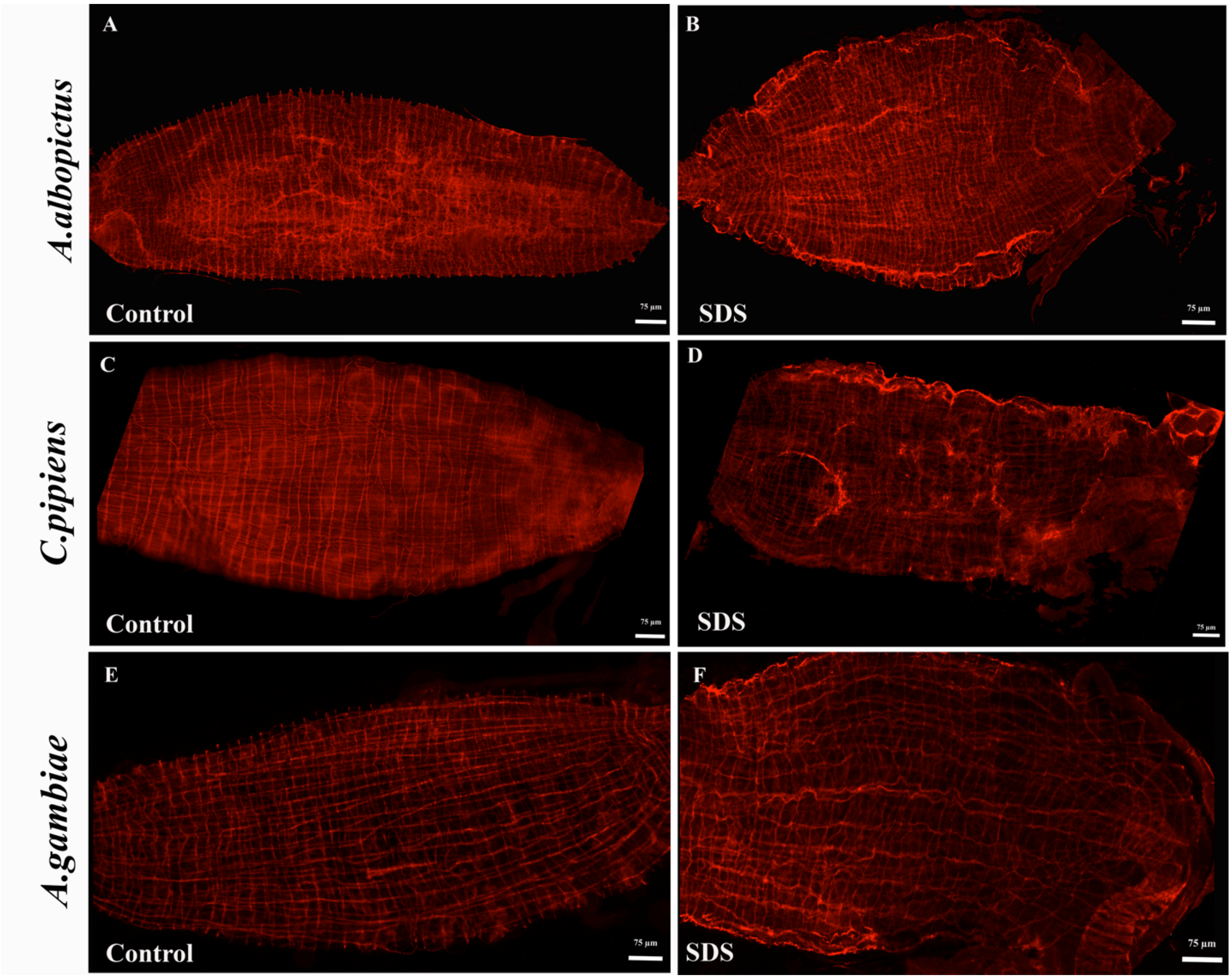
A.albopictus
C.pipiens
A.gambiae
A
B
C
D
E
F
Control
SDS
Control
SDS
Control
SDS
75 µm

Figure S3

Figure S3: SDS feeding affects the gut morphology in three mosquito species

Phalloidin staining of dissected guts shows that the guts of mosquitoes fed for 24 hours on sucrose supplemented with 2% SDS have an altered morphology (S3B, S3D and S3F) as compared to controls fed on sucrose (S3A, S3C and S3E). Phalloidin coupled to Alexa Fluor® 647, and was added for one hour at room temperature (1:500 in PBS-Triton 0.1%-BSA 1%).

A

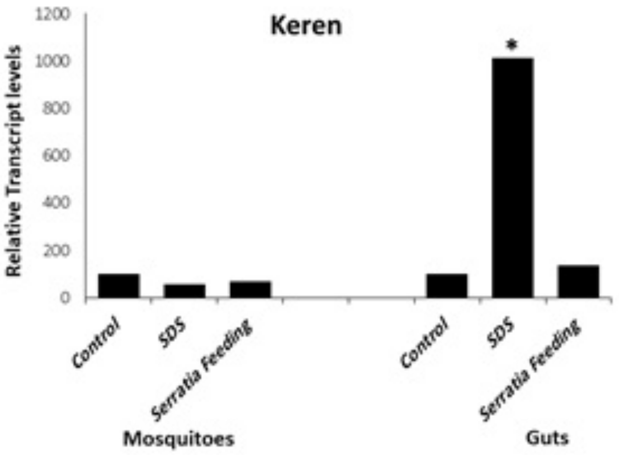
Keren
Relative Transcript levels
1200
1000
800
600
400
200
0
*
Control
SDS
Serratia Feeding
Control
SDS
Serratia Feeding
Mosquitoes
Guts

B

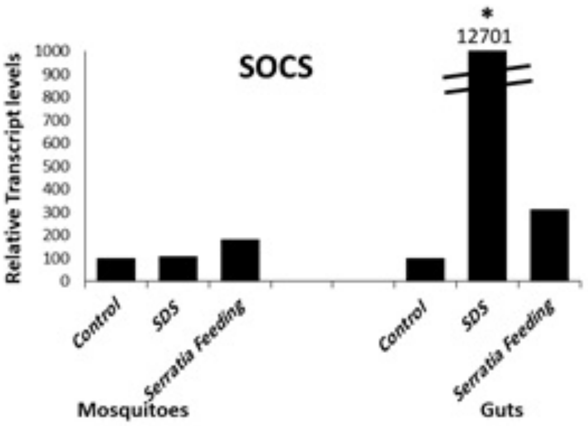
SOCS
Relative Transcript levels
*
12701
1000
900
800
700
600
500
400
300
200
100
0
Control
SDS
Serratia Feeding
Control
SDS
Serratia Feeding
Mosquitoes
Guts

Figure S4

Figure S4: Relative transcription levels of Keren and SOCS36E genes in *A. gambiae* after SDS or *S. marcescens* feeding

Feeding *A.gambiae* mosquitoes on 2% SDS-sucrose solution leads to a significant increase in the transcription of both Keren and SOCS36E genes in the gut (*P<0.05) but not in the whole mosquito as revealed by real time PCR. *S. marcescens* oral infection did not lead to statistically significant results. Real-time PCR was performed on BIORAD thermocycler (CFX 96 Real-time System, C1000). Ct values for target genes were normalized to Rps7 and compared to controls using the delta Ct method. Three independent experiments were averaged and unpaired t tests were performed.

Primers used wre as follow:

Socs Forward: 5'-GTTTTCCGTCTCCTTCCGCAAGTA-3'

Socs Reverse: 5'-CTTCGGTAGCGTCAGCTCGTTGAT-3'

Keren Forward: 5'-CTCGTCCTCCCAGTCCTACA-3'

Keren Reverse: 5'-TCGAACAAAACCAGGGTCTGA-3'

Rps7 Forward: 5'-TTCAACAACAAGAAGGCGATCA-3'

Rps7 Reverse: 5'-CTTGTACACCGACGCAAAAGTG-3'
